# TNF-alpha and metalloproteases as key players in melanoma cells aggressiveness

**DOI:** 10.1186/s13046-018-0982-1

**Published:** 2018-12-28

**Authors:** Stefania Rossi, Martina Cordella, Claudio Tabolacci, Giovanni Nassa, Daniela D’Arcangelo, Cinzia Senatore, Paolo Pagnotto, Roberta Magliozzi, Annamaria Salvati, Alessandro Weisz, Antonio Facchiano, Francesco Facchiano

**Affiliations:** 1 Department of Oncology and Molecular Medicine, Istituto Superiore di Sanità, ISS, viale Regina Elena 299, 00161 Rome, Italy; 2 Laboratory of Molecular Medicine and Genomics, Department of Medicine, Surgery and Dentistry ‘Scuola Medica Salernitana’, University of Salerno, Baronissi, SA Italy; 3 Laboratory of Molecular Oncology, Istituto Dermopatico dell’Immacolata, IDI-IRCCS, Rome, Italy; 4 Neurology B, Department of Neurological and Movement Sciences, University of Verona, Verona, Italy; 5 Genomix4Life srl, Baronissi, SA Italy

**Keywords:** Cancer, Cytokines, Inflammation, Malignancy, Metalloproteases, Cutaneous melanoma, Uveal melanoma, Proteomics, TNF

## Abstract

**Background:**

Melanoma aggressiveness determines its growth and metastatic potential. This study aimed at identifying new molecular pathways controlling melanoma cell malignancy.

**Methods:**

Ten metastatic melanoma cell lines were characterized by their proliferation, migration and invasion capabilities. The most representative cells were also characterized by spheroid formation assay, gene- and protein- expression profiling as well as cytokines secretion and the most relevant pathways identified through bioinformatic analysis were tested by in silico transcriptomic validation on datasets generated from biopsies specimens of melanoma patients. Further, matrix metalloproteases (MMPs) activity was tested by zymography assays and TNF-alpha role was validated by anti-TNF cell-treatment.

**Results:**

An aggressiveness score (here named Melanoma AGgressiveness Score: MAGS) was calculated by measuring proliferation, migration, invasion and cell-doubling time in10human melanoma cell lines which were clustered in two distinct groups, according to the corresponding MAGS. SK-MEL-28 and A375 cell lines were selected as representative models for the less and the most aggressive phenotype, respectively. Gene-expression and protein expression data were collected for SK-MEL-28 and A375 cells by Illumina-, multiplex x-MAP-and mass-spectrometry technology. The collected data were subjected to an integrated Ingenuity Pathway Analysis, which highlighted that cytokine/chemokine secretion, as well as Cell-To-Cell Signaling and Interaction functions as well as matrix metalloproteases activity were significantly different in these two cell types. The key role of these pathways was then confirmed by functional validation. TNF role was confirmed by exposing cells to the anti-TNF Infliximab antibody. Upon such treatment melanoma cells aggressiveness was strongly reduced. Metalloproteases activity was assayed, and their role was confirmed by comparing transcriptomic data from cutaneous melanoma patients (*n* = 45) and benign nevi (*n* = 18).

**Conclusions:**

Inflammatory signals such as TNF and MMP-2 activity are key intrinsic players to determine melanoma cells aggressiveness suggesting new venue sin the identification of novel molecular targets with potential therapeutic relevance.

**Electronic supplementary material:**

The online version of this article (10.1186/s13046-018-0982-1) contains supplementary material, which is available to authorized users.

## Background

Melanoma incidence and mortality are steeply increased in the last century [1–3]. Melanoma is the most aggressive skin cancer and the cutaneous form (Cutaneous Melanoma, CM) is the most common one. Recent data on novel pathways involved in melanoma development opened new opportunities to identify novel therapeutic targets [4, 5], nevertheless additional key players underlying melanoma onset and progression need to be identified. Indeed, further elucidation of the molecular mechanisms underlying melanoma malignancy is expected to improve prognostic assessment and therapeutic options. The role of altered RAS/BRAF/MEK/ERK pathway in melanoma pathogenesis and progression is well known [6]. Mutated BRAF^V600E^ represents a major target in the current therapeutic strategies, despite the fact that more than half of melanomas do not harbor this mutation. Melanoma highly aggressive behavior depends on migration, invasion, proliferation of metastatic cells and on their ability to promote angiogenesis [7]. Invasive CM cells metastasize changing cytoskeletal organization and modifying the interaction with the extracellular matrix (ECM) and the surrounding stromal cells. During the vertical growth phase, primary tumor cells invade the dermis [8], via a cross-talk with the neighboring microenvironment [9, 10]. Proteolysis in the pericellular and stromal compartments, in fact, exerts a key role in the invasion process and it is well know that several protease, such as matrix metalloproteases (MMPs), are mediators of melanoma development [11]. On the other hand, MMP-9 activation was associated with cancer growth and dissemination [12], its role in cutaneous melanoma was reported and its activation, mediated by NF-κB, was associated with the BRAFV600E mutation status [13]. Another recent study reports the correlation of MMP-9 hypermethylation with its overexpression in melanoma [14] indicating novel molecular mechanisms underlying the MMPs activity and their modulatory role in melanoma aggressiveness.

Different molecules play a role in cancer progression, including chemokines and their receptors, as well as cytokines and growth factors [15]. Melanoma cells often express variable levels of cytokines and cytokine receptors at different stages of disease progression. Interleukin (IL-)1β, IL-6 and IL-8, for example, are known to be important drivers of cell proliferation and melanoma progression [16, 17]. Nevi and thin primary melanomas (less than < 1 mm of thickness) express low levels of IL-8, tumor necrosis factor-alpha (TNF-α), transforming growth factor-beta (TGF-β) and c-kit [18]. On the contrary, primary melanomas at more advanced stage (> 1 mm of thickness) show up-regulation of IL-1α, IL-1β, IL-8, TNF-α, TGF-β and granulocyte-macrophage colony stimulating factor (GM-CSF). TGF-β is considered a marker of melanoma metastatic spreading [18]. Moreover, a link between high levels of TNF-α and increased risk of tumor formation and development has been described in vivo [19]. An additional study in a murine model shows that more aggressive tumors express lower levels of TNF-α and other inflammatory cytokines, as determined by qRT-PCR analyses [20], confirming previously reported controversial role of TNF-α [21]. This suggests that melanoma development and progression is a complex process based on a well-organized interplay of intrinsic proliferation ability combined to the immune and angiogenic response, as coordinated action of several cell types. Cytokines controlling inflammation and immune cells can influence host immune response and melanoma cells can activate and/or reshape the surrounding environment to secrete factors mediating metastatic progression. Furthermore, melanoma cells can secrete inhibitory modulators and thereby arrest recognition and maturation of effector immune cells [22] or other signals affecting cancer cells microenvironments [23]. Therefore, several different mechanisms control CM ability to rapidly grow, invade and disseminate metastases in other tissues and organs. This highlights the importance of microenvironment and immune response to CM, as strongly influenced by intrinsic characters of primary melanoma cells.

Understanding such multifaceted functional interactions, involving different cell types and molecules, requires to integrate information gathered by different analytical approaches [24–26]. The aim of the present study was to investigate intrinsic factors affecting human melanoma cell aggressiveness, investigating at different levels human melanoma cells that hold highly different malignancy grade. The study led to the novel identification of molecular targets and functional pathways likely responsible of the melanoma aggressive phenotype.

## Methods

### Experimental design and cell culture

The aggressive phenotype of CM is responsible for the very poor prognosis of this disease as in advanced or in recurrent cases [1]. CM aggressiveness has been associated with its mutational state (e.g. to bear or not a V600E BRAF mutation, alone or together with others), and also with the anatomical site where the primary tumor occurs or, in addition, with the immunological status of patients observed in different cases of patients receiving immunosuppressive therapies [27, 28]. Beside these considerations, one crucial question is whether such melanoma cell aggressiveness may be explained also by the presence of any intrinsic behavior of melanoma cell itself.

Human melanoma cells expressing different aggressiveness were therefore compared under very similar culture conditions (melanocytes were not included in this study as a control, due to the highly different culture medium used for their in vitro culture; see Additional file 1: Table S1).

Ten human cell lines were used, as summarized in Table 1. Human metastatic cutaneous melanoma cell lines used were: SK-MEL-28, A375 and A375M (purchased and authenticated from the American Type Culture Collection, ATCC, Manassas, VA), Mel-397 (kindly supplied by Dr. Stefania D’Atri, IDI-Roma), SK-MEL-110 [29], Preyer, SK-MEL-30 and MEWO (kindly provided by Dr. Tobias Haas, ISS, Rome) [30]**,** MEL501 and ME665 (kindly provided by Dr. Francesca Urbani, ISS-Rome). Preliminary experiments included additional human melanoma cell lines such as WM-115, SK 120 and SK 147 from established culture [31] and human uveal melanoma cell lines as (92.1, OMM1, OMM 2.5 and UPMM3, kindly provided by Dr. Giovanna Angelini, IST, Genova, Italy) [32]**.** All cell lines were cultured in the specific standard conditions following the manufacturer’s instructions or as previously reported [33, 34]. In detail, A375, Preyer, SK-MEL-30, MEWO, MEL501 and ME665 were propagated in complete Dulbecco’s modified Eagle’s medium (DMEM; Hyclone, South Logan, UT) supplemented with 10% fetal bovine serum (FBS, HyClone), 2 mM L-glutamine and 100 IU/ml penicillin/streptomycin (Invitrogen, Carlsbad, CA) in humidified 5% CO_2_ atmosphere, at 37 °C for the specified time and, when required, under serum deprivation. Mel 397, SK-MEL-28 and SK- Mel-110 were cultured in RPMI 1640 (Hyclone) 10% Fetal Calf Serum (FCS), 2 mM L-glutamine, and 100 IU/ml penicillin/streptomycin and, when required, under serum deprivation.

**Table 1:** Human melanoma cell lines used in the present study, with reference to the mutational state

	Mutant Gene	Gene Sequence	Protein Sequence	PubMed ID
A375	BRAF CDKN2A CDKN2A	c.1799 T > A c.181G > T c.205G > T	p. V600E p.E61* p.E69*	16,801,397 7,923,152
A375M	BRAF	c.1799 T > A	p. V600E	25,684,511
ME 665	NRAS	c.182A > G	p.Q61R	8,032,213
Mel 397	BRAF		p. V600E	
MEL 501	NRAS BRAF	c.35G > A c.1799 T > A	p.G12D p.V600E	24,838,835 15,467,732
MeWo	CDKN2A TP53 TP53	c.238C > T c.772G > A c.949C > T	p.R80* p.E258K p.Q317*	7,478,563 11,096,420
Preyer	n/a	n/a	n/a	n/a
SK-MEL-110	TP53	n/a	n/a	16,267,831
SK-MEL-28	BRAF TP53 CDK4	c.1799 T > A c.434 T > C c.70C > T	p. V600E p. L145R p.R24C	16,170,021 23,856,246 23,856,246
SK-MEL-30	NRAS CDKN2A	c.181C > A c.341C > T	p.Q61K p.P114L	10,766,161 8,895,759

The asterisks refer to the mutations as reported onto the ATCC catalog

### Proliferation assay

Proliferation assays were carried out as previously described [26]. Briefly, cells (6 × 10^4^ cells/well) were seeded in 6-well plates and grown for24 h in the presence of 10% FCS and then grown for 24 h and/or 48 h in serum-free medium. Subsequently, cells were washed using phosphate buffer saline (PBS w/o Ca^2+^/Mg^2+^), harvested with trypsin/EDTA and counted with a Neubauer modified chamber as previously reported [26]. To investigate the cell-cell signaling and interaction, additional proliferation assays at three different cell-densities were carried out, namely high (9x10^4^cells/well), intermediate (6 × 10^4^ cells/well) and low (3 × 10^4^ cells/well), following the same experimental procedure reported above. All experiments were performed at least 3 times in duplicate.

### Spheroid formation assay

Spheroid formation assay was performed as previously described [35]. Briefly, A375 and SK-MEL-28 cells (5000cells/ml) were plated in ultralow attachment plates (Corning, NY, USA) in a serum-free medium as described [36]. Primary spheroids were collected after 7/14 days, dissociated into single cell suspension, counted and plated again in another ultralow attachment plate at 1000 cells/ml density. After 7 days, secondary spheroids were photographed, dissociated into single cells and counted.

### Cell migration and invasion assays

Cell migration assay was carried out by growing cells to confluence in 12-well plates and wounds were made with a sterile plastic tip as described [37]. Melanoma cells were incubated for 24 h in the absence of FCS and photographed under microscope at time 0 and after 24 h. The number of migrating cells was quantified by Image J software (NIH: https://imagej.nih.gov/ij/) and expressed as a percentage of control. Cell invasion ability was tested using a commercial Transwell system (24-well plates, 8.0 μm pore size, Corning, NY, USA). Transwell upper inserts were coated with 0.1 ml of BioCoat™ Matrigel™ and incubated overnight at 37 °C, then 0.2 ml warm (37 °C) serum-free medium was added to melanoma cells (2 × 10^5^) seeded into the upper wells of 24-well Transwell plates on Matrigel. Lower wells contained complete medium with 50% FBS. After 24 h incubation in a humidified 5% CO_2_atmosphere, the upper well content (non-invading cells) was removed, the inserts were washed with PBS and cells were fixed with absolute ethanol. The invasion chambers were processed following the manufacturer’s protocols, and cells were stained with 5% GIEMSA as described [38]. Cells were counted under phase-contrast microscopy. To confirm data obtained by the Transwell model, invasiveness of melanoma cells was also evaluated in Boyden Chamber assay as described [39], with polyvinylidene difluoride (PVPF) membrane (8 μm pore size, Costar, Cambridge, MA). The membrane was fixed in absolute ethanol and stained with 5% GIEMSA. After removal of non-migrating cells in the upper side of the membrane, the number of invasive cells was calculated as the mean of 8 microscopy-fields.

### Calculation of the aggressiveness score

In order to develop a tool able to quantify melanoma cell malignancy, the Melanoma cell AGgressiveness Score (MAGS) was calculated. Such score was a quantitative parameter obtained from the combination of proliferation, migration and invasiveness ability of each investigated melanoma cell. MAGS score was calculated using the following algorithm:

$$ \mathrm{MAGS}=\frac{\mathrm{Growth}\times \mathrm{Migration}\times \mathrm{Invasion}}{\mathrm{Doubling}\ \mathrm{time}} $$

were Growth is the percentage of proliferation after 24 h, Migration is the percentage of plate surface covered by migrated cells after 24 h, Invasion is the percentage of cells passing through the Transwell filter after 24 h (all such parameters were compared to time zero), Doubling time is the time cells use to double their number, computed according to the last square fitting exponential method expressed in hours.

### Programmed cell death analysis

Cells cultured in 6-well plates (Corning, NY, USA) were harvested, taking into account both floating and attached cells, and fixed in 80% cold ethanol. Fixed cells were washed and incubated with10 μg/ml propidium iodide (PI) and 200 μg/ml ribonuclease A (RNAse A, Thermo Fisher, MA,USA) as previously described [31]. The relative DNA content and cells distribution in cell cycle phases were determined with both FACScan Becton Dickinson Instrument (Becton Dickinson, CA, USA) and the FACS Diva software (5.0.3 version) as previously described [40].

### Cytokines and growth factor analysis

Cytokines and growth factors were measured by xMAP multiplex technology. Bio-Plex Pro human cytokine 27-plex panel (Bio-Rad Laboratories, Hercules, CA) allowed to measure the following analytes: IL-1Ra, IL-1β, IL-2, IL-4, IL-5, IL-6, IL-7, IL-8, IL-9, IL-10, IL-12(p70), IL-13, IL-15, IL-17, TNF-α, IFN-γ, Macrophage Inflammatory Protein (MIP)-1α, MIP-1β, Eotaxin, Monocyte Chemoattractant (MCP)-1 (CCL2), Granulocyte Colony stimulating factor (G-CSF), GM-CSF, Basic Fibroblast growth factor (FGF-2), Vascular endothelial growth factor (VEGF), Interferon gamma-induced protein 10 (IP-10), Regulated on Activation, Normal T cell Expressed and Secreted (RANTES or CCL5), and Platelet-derived growth factor (PDGF)-BB. Conditioned media were collected, centrifuged and four-fold concentrated using a centrifugal filter unit trough microporous membrane 3 kDa cut-off (Centriprep YM-3, NMWL 3 kDa, Merck KGaA, Darmstadt, Germany)**.** Proteins concentration was then measured using the Bradford assay (Bio-Rad) according to manufacturer’s instructions. Additional experiments were carried out by measuring cytokines level into cell lysates, prepared as previously reported [26]. The analysis was carried out using 50 μl of sample. After incubation with antibodies-activated magnetic beads, samples were washed using a Bio-Plex Pro™ Station (Bio-Rad). The quantification was carried out on a Luminex X200 platform (Bio-Plex® Bio-Rad), a Bio-Plex Manager Software version 6.1 and results were expressed as pg/ml/mg of protein. Protein concentration was evaluated according to the Bradford assay (Bio-Rad). Each sample was analysed at least three times in duplicate. Data are expressed as mean ± standard deviation (SD).

### RNA purification, gene expression microarray and data analysis

After medium removal, cells were harvested and lysed in TRIzol reagent (Invitrogen Corporation, Carlsbad, CA, USA). Total RNA was then isolated from the samples following the manufacturer’s instructions. Before use, RNA concentration in each sample was assayed with NanoDrop 2000C spectrophotometer (NanoDrop, Thermo Scientific, Rockford, IL) and its quality was assessed with an Agilent 2100 Bioanalyzer with the Agilent RNA 6000 nano kit (Agilent Technologies, Santa Clara, CA) as previously described [41]. mRNA microarrays analyses were performed using 500 ng of total RNA as starting material for the synthesis of cDNA and biotinylated cRNA, according to the Illumina Total Prep RNA Amplification Kit protocol (Ambion, Austin, TX,). For each sample, 750 ng of cRNA were hybridized on Illumina HumanHT-12 v 4.0 BeadChips (Illumina Inc.) as described earlier [42] and subsequently scanned with the Illumina iSCAN. Data analyses were performed with Genome Studio software version 2011.1 (Illumina Inc.). Data were normalized with the quantile algorithm, and genes were considered detected if the detection *p-value* was less than 0.01. Statistical significance was calculated with Illumina DiffScore, a proprietary algorithm that uses the bead standard deviation to build an error model. Only genes with a DiffScore of - ≤30 or ≥ 30, corresponding to a *p-value* of 0.001, were considered as statistically significant by comparing all values obtained in A375 cells compared to the SK-MEL-28 values. Raw and quantile normalized microarray data have been deposited, in a format complying with the Minimum Information about a Microarray Gene Experiment guidelines of the Microarray Gene Expression Data Society, in the EBI Array- Express database (www.ebi.ac.uk/arrayexpress) with accession number E-MTAB-4212.

### Mass spectrometry and proteomic analyses

Postnuclear cell lysates were prepared and denatured by using the three denaturation treatment (TRIDENT) protocol as previously described [43] and were run in a 4–15% polyacrylamide gel [44]. For protein identification, the whole lane of the gel was cut in several pieces, proteins were reduced, alkylated and digested overnight with bovine trypsin sequencing grade (Roche Applied Science, Monza, IT) according to a published protocol [45]. The peptide mixtures were analyzed by nano-reversed-phase liquid chromatography tandem mass spectrometry (nRP-LC-MS/MS) using an HPLC Ultimate 3000 (DIONEX, Sunnyvale, CA) connected on line with a linear Ion Trap (LTQ, Thermo Electron, San Jose, CA) as described [44]. Data acquisition and analysis was performed as previously reported [43]. Data were searched with 1.5 Da and 1 Da tolerance respectively for precursor and fragment ions. A peptide was considered legitimately identified when it achieved cross correlation scores of 1.5 for [M + H]1+, 2.0 for [M + 2H]2+, 2.5 for [M + 3H]3+, and a peptide probability cut-off for randomized identification of *P* < 0.001.

### Bioinformatic analyses

Genes and proteins lists obtained from the above reported analyses were analyzed using Ingenuity Pathway Analysis Software (IPA, Ingenuity® Systems, www.ingenuity.com) as previously described [26, 46]. In details, it refers to a proprietary knowledge base (Ingenuity Pathways Knowledge Base) annotating molecules, biological interactions and functional properties. IPA Functional Analysis on “molecular and cellular functions” category and Canonical Pathway investigation were performed calculating the likelihood that the association between our transcription dataset and a specific function or pathway is due to random choice, and it is expressed as a *-value* calculated using the right-tailed Fisher Exact Test. In network generation, each differentially expressed transcript identifier was uploaded and mapped to its corresponding object in Ingenuity Knowledge Base to algorithmically generate molecular networks based on their connectivity. The networks were scored according to a numerical value considering the number of dataset molecules and the network size as well as the total number of input transcript in the dataset and the total number of molecules in the Ingenuity Knowledge Base that could potentially be included in the networks. The network Score is based on the hypergeometric distribution and is calculated with the right-tailed Fisher Exact Test. The upstream regulator analysis is based on prior knowledge of expected effects between transcriptional regulators and the differentially expressed transcript dataset of target genes by using information in Ingenuity Knowledge Base. For each potential Upstream Regulator (“UR”) two statistical measures, an overlap *p-value* and an activation *z-score* were computed. The overlap p-value calls likely URs based on significant overlap between dataset genes and known targets regulated by a UR. The activation *z-score* is used to infer likely activation states of upstream regulators based on comparison with a model that assigns random regulation directions. Under ideal circumstances (the “un-biased” case described below) the activation z-score can also be used to predict upstream regulators independently from the overlap p-value, based on significant pattern match of up/down regulation.

Pathways predicted as potentially involved in melanoma cell aggressiveness by IPA analyses were further analyzed in GEO database (https://www.ncbi.nlm.nih.gov/sites/GDSbrowser). Proteins identified by proteomic analysis were analyzed by the Database for Annotation, Visualization and Integrated Discovery (DAVID software, http://david.abcc.ncifcrf.gov/) that provides a comprehensive set of functional annotation tools for investigators to understand biological meaning behind a large list of proteins.

### Validation of the identified molecular pathways

Semi-confluent cells were harvested, plated in medium with 10% FBS in 6-well plates at 3 × 10^4^ cells/well density. After 24 h, media were removed, cells were washed with PBS and medium was replaced with serum-free medium. Cells, starved for 24 h, were incubated with Infliximab antibody (IFX) (Janssen Biothec, Inc., USA) at different concentrations (10, 100, and 1000 ng/ml) for 24 h, then cells were washed, harvested with trypsin/EDTA and counted with Neubauer modified chambers. All experiments were carried out at least 3 times in duplicate. Metalloprotease involvement was assayed by gelatin zymography, MMP-2 activity was analyzed in melanoma cells conditioned medium according to a published procedure [47] with few modifications [48].

### Statistical analysis

All experiments were carried out at least three times and the results were expressed as mean ± standard deviation (SD). Data were analyzed by the two tails *t*-Student test. Differences were considered significant when *P* < 0.05.

## Results

### Melanoma cell AGgressiveness score (MAGS): Cell proliferation, migration and invasion studies

**Fig. 1:**
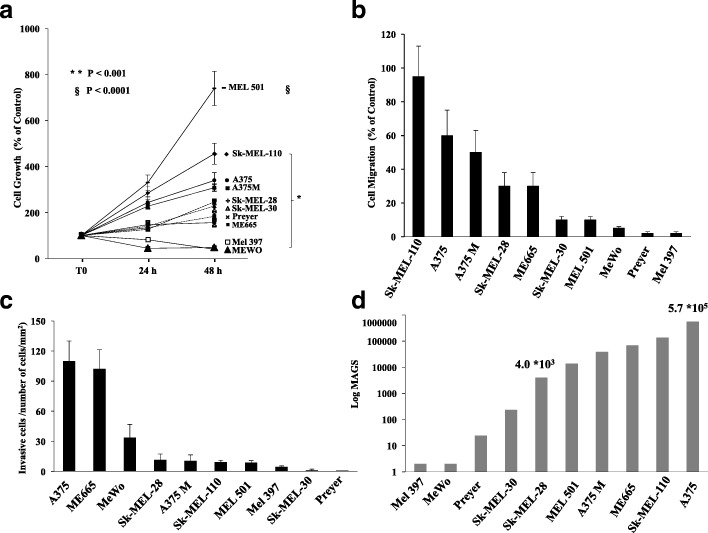
Characterization of melanoma cell aggressiveness: all the cell lines were analysed simultaneously using the same experimental procedures. Evaluations and quantifications were assessed by two different operators in blind. **a** Growth rate after 24 and 48 h of serum starvation. After serum deprivation, cells were incubated for 24 and 48 h and then they are harvested and counted. Cell counts for each cell line was: 61729 for SK-MEL-110, 57,250 for Mel 397, 46,171 for SK-MEL-30, 57,000 for Preyer, 61,333 for A375M, 60,212 for A375, 61,143 for SK-MEL-28, 60,600 for Mewo, 60,500 for Mel 501, 60,500 for Me 665. The data represent the mean ± SD of three experiments carried out in triplicate (statistical significance versus control: **P < 0.001; § *P < 0.0001*). **b** Migration ability of melanoma cell lines. The scratch test on confluent cells were performed for 24 h. **c** Invasion analysis of melanoma cells for 24 h. The invasion capability is expressed as number of cells per mm^2^ of filter. **d** Aggressiveness index (MAGS index calculated as reported in Methods), to cluster melanoma cell lines accordingly to their malignancy: a combination of growth, invasion and migration rates was used to get such aggressiveness index

Human cutaneous melanoma cell lines, summarized in Table 1, were used to investigate and compare their aggressive phenotype. For proliferation assays, cells were grown in serum-free medium [49] and cell number was measured at 24 and 48 h of serum deprivation and expressed as % number vs Time 0 (Fig. 1a).Different growth rates were observed in the 10 different cell lines; they were then clustered in three main groups, namely: high proliferation rate (SK-MEL-110, A375, A375M, MEL501), low proliferation rate (ME665, SK-MEL-30, Preyer, SK-MEL-28) and very low proliferation rate (Mel 397 and MeWo) cells (Fig. 1a). The scratch test was then carried out to measure migration of the 10 melanoma cell lines, under serum deprivation conditions. Results are depicted in Fig. 1b. SK-MEL-110, A375 and A375M cells showed the highest migration rate, while SK-MEL-28, ME665, SK-MEL-30, MEL501 cells showed intermediate migration ability; Preyer, MeWo and Mel 397cells showed very low migration potential. Invasion was then analyzed and A375 and ME665 showed the highest invasiveness potential while SK-MEL-30 and Preyer cell lines showed an almost absent invasion ability (Fig. 1c). Then by combining proliferation, migration and invasion rates, including the doubling time, the Melanoma cell AGgressiveness Score (MAGS) was calculated for each cell line, as reported in Methods (Fig. 1d). Cells with very low MAGS were excluded for further omics studies, due to difficulties to obtain cell lysates with a good protein recovery. Therefore, to recapitulate these differences, two cell lines were selected, namely A375 as the most aggressive and SK-MEL-28 as the less aggressive. Interestingly, A375 and SK-MEL-28 are among the best characterized human melanoma cells lines in literature even from the mutational and genetic point of view.

### A375 and SK-MEL-28 characterization

**Fig. 2:**
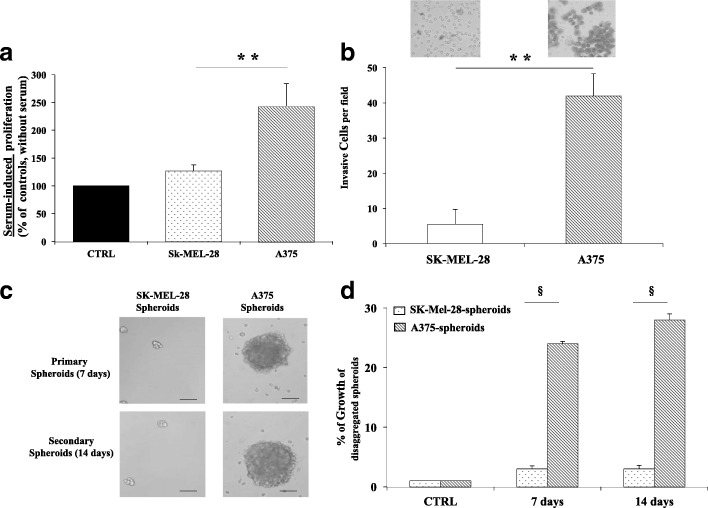
Characterization of A375 and SK-MEL-28, respectively, the most and the less aggressive model of human melanoma cell lines. **a** Cell proliferation after 24 h of serum deprivation. A375, under an extreme growing condition, showed a significantly (**P < 0.001) higher growth rate than SK-MEL-28. Data are expressed as percent of 61,143 cells for SK-MEL-28 and 60,212 cells for A375 and represent the mean ± SD of three experiments carried out in triplicate. **b** Invasion ability of melanoma cell lines by Boyden chamber assay. A375 were significant able to invade in respect to SK-MEL-28 (**p < 0.001); in particular, a mean of 42 and 5 cell per field were counted respectively). **c** Microphotographs showing forming spheroid capability. **d** The panel indicates the quantification of total cell number forming A375 and SK-MEL-28 spheroids (§ *p < 0.0001*)

A375 and SK-MEL-28 cells proliferation was then evaluated in the presence of serum; under such conditions A375 cell line confirmed to grow at a much higher rate as compared to SK-MEL-28 (Fig. 2a)*.*In addition, serum-induced invasion of A375 was found to be much higher than SK-MEL-28 (*p < 0.001*) (Fig. 2b).The spheroid colony formation capability assay (melanosphere forming assay) was then carried out as described [35]. As shown in Fig. 2c and d, A375 cells showed a significantly (*p < 0.0001*) higher ability to form melanosphere (A375-spheroids) as primary and secondary spheroids as compared to SK-MEL-28 (SK-MEL-28-spheroids) after both 7 and 14 days of growth, respectively. Total spheroids were then dissociated into single cell suspension and counted with similar results. Since this assay allows to evaluate the stem traits of tumor cells that is related to resistance to extreme conditions and treatments, these experiments confirmed that the biological features of the selected cell lines, under our experimental conditions, were strikingly different, with the A375 showing a more aggressive phenotype compared to SK-MEL-28. Proliferation of the selected cell lines was analyzed in deprivation serum condition and at three different cell densities. As depicted in Additional file 1: Figure S1 the two cell lines grow at a similar time-dependent rate, at intermediate (intermediate panel) and high (lower panel) density, while at the lowest cell density (upper panel) SK-MEL-28 were unable to grow differently from A375. This suggested that cell-cell signaling and/or secretory signals related to the cell-density may be at least in part involved in their aggressive phenotype. Interestingly, under low cell density conditions, SK-MEL-28 cells showed the smallest growth within the 10 cell lines tested (data not shown). The aggressive phenotype was also evaluated as sensitivity to serum-starvation and apoptotic stimuli. A375 confirmed their higher malignancy since resulted to be more resistant to both serum-starvation and apoptotic stimuli when compared to SK-MEL-28 (see Additional file 1: Figure S2).

### Transcriptome analysis in differently aggressive melanoma cell lines

**Fig. 3:**
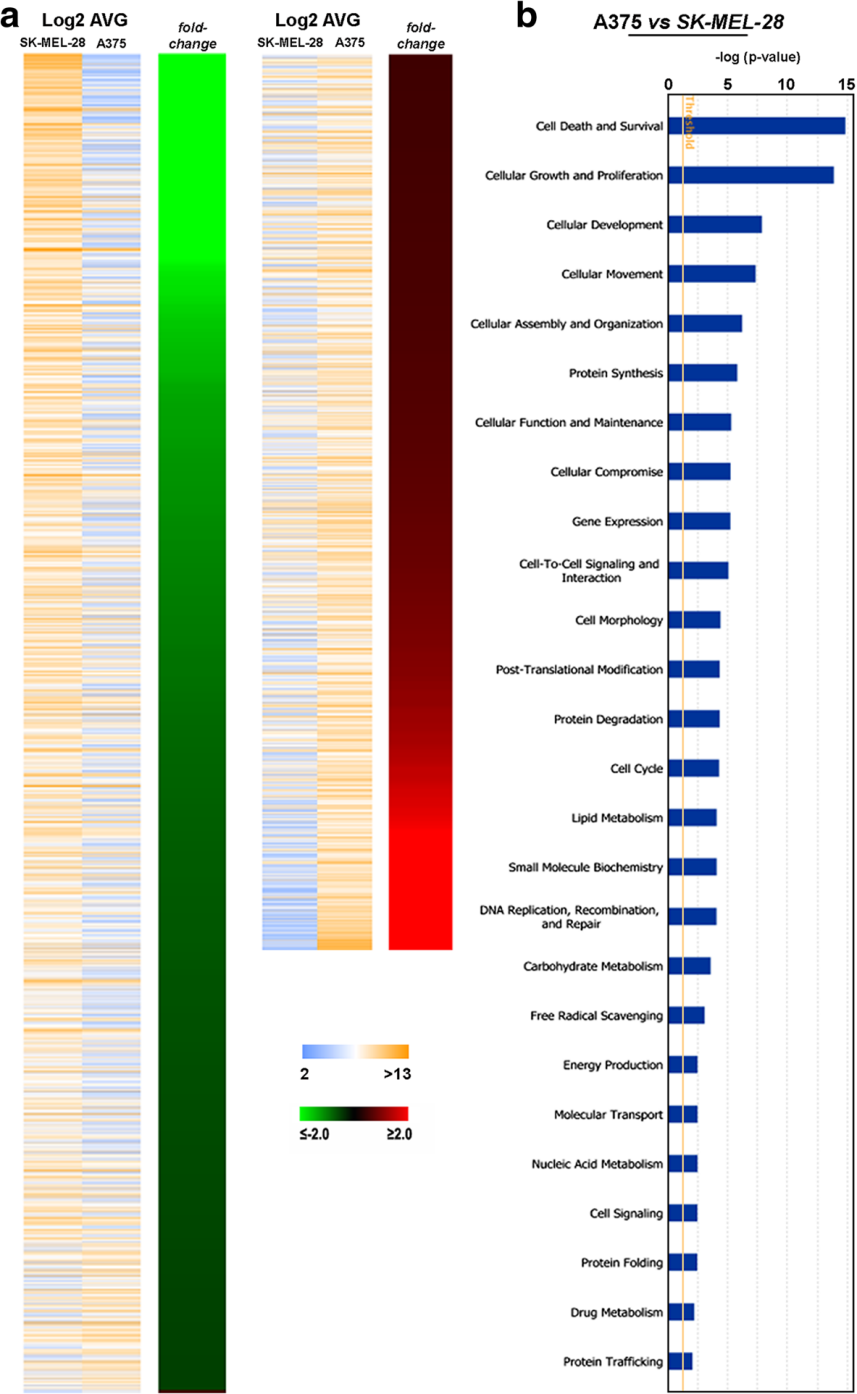
Aggressiveness driven de-regulation of transcriptome in melanoma cells behaving and functional analyses of the results. **a** Heat map of differentially expressed transcripts in A375 *vs*SK-MEL-28 human melanoma cells according to log2 AVG signals (left) and *fold-change* (right). **b** Graph showing most significantly enriched molecular functions identified by the IPA analysis. Each histogram reports the –log of the *p*-value (Fisher’s exact test) for each molecular function. The straight orange lines mark the significance p-value threshold (0.05)

To better investigate the molecular basis of the observed different growth/invasive phenotype, global gene expression profile was performed from A375 and SK-MEL-28 cells under the same culturing conditions. Out of the 2973 transcripts found differentially expressed between the two cell lines, 1513 resulted down-regulated and 1460 up-regulated in A375 vs SK-MEL-28 (FC |1.5|, *p*-value < 0.001) as reported in Additional file 1: Table S2. The heat map reported in Fig. 3a shows the fold change difference of each gene obtained comparing the two cell lines, highlighting the strong difference in their gene expression profile. The Ingenuity Pathway Analysis (IPA) conducted on the differentially expressed genes in A375 vs SK-MEL-28 cells revealed that such transcripts are involved in 26 key “molecular and cellular functions categories” (Fig. 3b and Additional file 1: Table S3a) such as Cell Death and Survival, Cellular Growth and Proliferation, Cellular Development, Cellular Movement, Cellular assembly and organization, Cell-To-Cell Signaling and Interaction. The identified genes fall in several “canonical pathways” (see Additional file 1: Table 3b) and 25 “top networks” (Additional file 1: Table 3c) mostly associated with inflammation, cell growth and proliferation and cell movement.

### Identification and validation of the upstream regulators

The IPA was then carried out to predict the upstream regulators of the genes reported in Fig. 3a. This analysis predicts, among others, MMP2, TNF and IL-6 (Additional file 1: Figure S3, S4 and S5) as strong upstream modulators of the transcriptome changes observed. We then aimed at validating such predictions, as reported below.

### Validation of metalloproteinase 2 (MMP-2) expression and activity

**Fig. 4:**
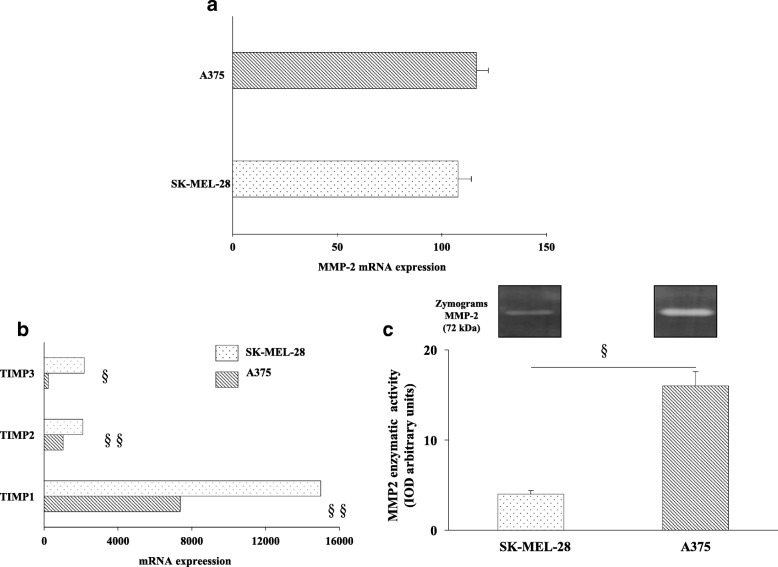
**a** Weak, not significant, MMP2 mRNA up-regulation in A375 compared to SK-MEL-28. **b** Highly significant (§§*p < 0.00001*; §*p < 0.0001*) down-regulation of Tissue Inhibitor of Metalloproteinases (TIMP) **c** The MMP2 activity evaluated by a gelatin zymography confirming the higher aggressiveness of A375 cells compared to SK-MEL-28

MMP2 mRNA expression was measured by RT-PCR and we found to be similar in A375 and SK-MEL-28, also confirming the transcription profiling data achieved by a different technological platform (Illumina)(Fig. 4a). Metalloproteinases activity is regulated by Tissue Inhibitor of Metalloproteinases (TIMPs), therefore TIMPs mRNA expression was analyzed and was found to be significantly downregulated in the most aggressive cell line compared to the less aggressive (*p < 0.001*) (Fig. 4b). As functional validation of these findings, the MMP-2–related enzymatic activity was then measured under serum starvation in A375 and SK-MEL-28 conditioned media by gelatin zymography. The evaluation of the integrated optical density (IOD) of zymograms confirmed that MMP-2 activity was 4-fold higher in A375 conditioned media than in SK-MEL-28 conditioned media (Fig. 4c) (*p < 0.001*). These data definitely confirm the strong involvement of MMP-2 enzymatic activity to explain different aggressiveness in the two melanoma cell models.

### Validation of TNF-α and IL-6 expression

To validate the IPA analysis regarding TNF-αand IL-6, the expression levels of human cytokines and growth factors secreted in the A375 and SK-MEL-28 growth media was measured. Table 2 shows that several cytokines are differently expressed and among these, TNF-α and IL-6. Namely, PDGF-BB, IL-1β, IL-9, IP-10, IL-8, IL-1ra and G-CSF resulted significantly down-regulated in A375 as compared to SK-MEL-28, while IL-6, IL-12, EOTAXIN, RANTES, INF-γ, TNF-α and VEGF were significantly up-regulated in A375 as compared to SK-MEL-28 (see Table 2). IPA analysis was then carried out on quantitative cytokines expression data. Table 3 reports the “Disease and Function”, “Pathways” and “Network” found significantly affected, confirming transcriptomic data analysis. The molecular mechanisms underlying the increased TNF pathway were then investigated. The levels of mRNA-TNF receptors were evaluated as potentially able to interfere with their expression and found not significantly modified (data not shown).

**Table 2:** Cytokines Levels in human melanoma cell lines by Luminex analysis

Cytokines	SK-MEL-28 (pg/ml/mgProt)	A375 (pg/ml/mgProt)	*p*-Value	A375 vs SK-MEL-28
PDGF-bb	5.79 ± 2.5	0.00 ± 0.00	0.0491	down-regulated
IL-1β	5.69 ± 3.59	1.98 ± 1.06	0.0001	down-regulated
IL-9	7228.00 ± 1003.00	1310.91 ± 446.08	0.0121	down-regulated
IP-10	83.44 ± 16.52	2.56 ± 07	0.0011	down-regulated
IL-8	322.20 ± 136.72	64.34 ± 13.87	0.0314	down-regulated
IL-1ra	80.35 ± 4.19	33.93 ± 16.17	0.0086	down-regulated
G-CSF	81.68 ± 14.20	22.94 ± 19.73	0.0138	down-regulated
IL-6	1.06 ± 0.04	5.86 ± 2.33	0.0235	up-regulated
IL-12	4.53 ± 2.59	16.38 ± 1.56	0.0008	up-regulated
Eotaxin	0.07 ± 0.02	54.67 ± 8.47	0.0000	up-regulated
RANTES	10.26 ± 1.62	234.04 ± 80.47	0.0015	up-regulated
IFN-γ	29.91 ± 1.23	348.61 ± 62.18	0.0009	up-regulated
TNF-α	1.09 ± 1.78	23.48 ± 3.46	0.0007	up-regulated
VEGF	2021.13 ± 82.71	2929.27 ± 1180,89	0.0037	up-regulated

Cytokines levels up- and down-regulation expressed in A375 compared to SK-MEL-28

**Table 3:** IPA analysis of quantitative cytokines level expression

IPA ANALYSIS		*p*-value	
Disease and Function	Cell-To-Cell Signaling and Interaction	8,54E-36	
	Inflammatory Response	4,1E-34	
	Cellular Growth and Proliferation	7,23E-34	
	Tumor Morphology	8,19E-24	
	Cell Signaling	1,18E-20	
	Molecular Transport	1,18E-20	
	Cellular Function and Maintenance	1,52E-18	
	Cell Morphology	1,48E-12	
	Cellular Assembly and Organization	1,05E-10	
	Protein Synthesis	7,14E-10	
		*p*-value	z-score
Pathways	Colorectal Cancer Metastasis Signaling	1,73E-02	2000
	PPAR Signaling	4,44E-02	1000
	LXR/RXR Activation	4,13E-02	0,447
	Dendritic Cell Maturation	4,14E-02	0,378
			Score
Network	Cell-To-Cell Signaling and Interaction		30
	Cardiovascular System Development and Function/Tissue morphology		15

### Proteomic analysis by mass spectrometry

**Fig. 5:**
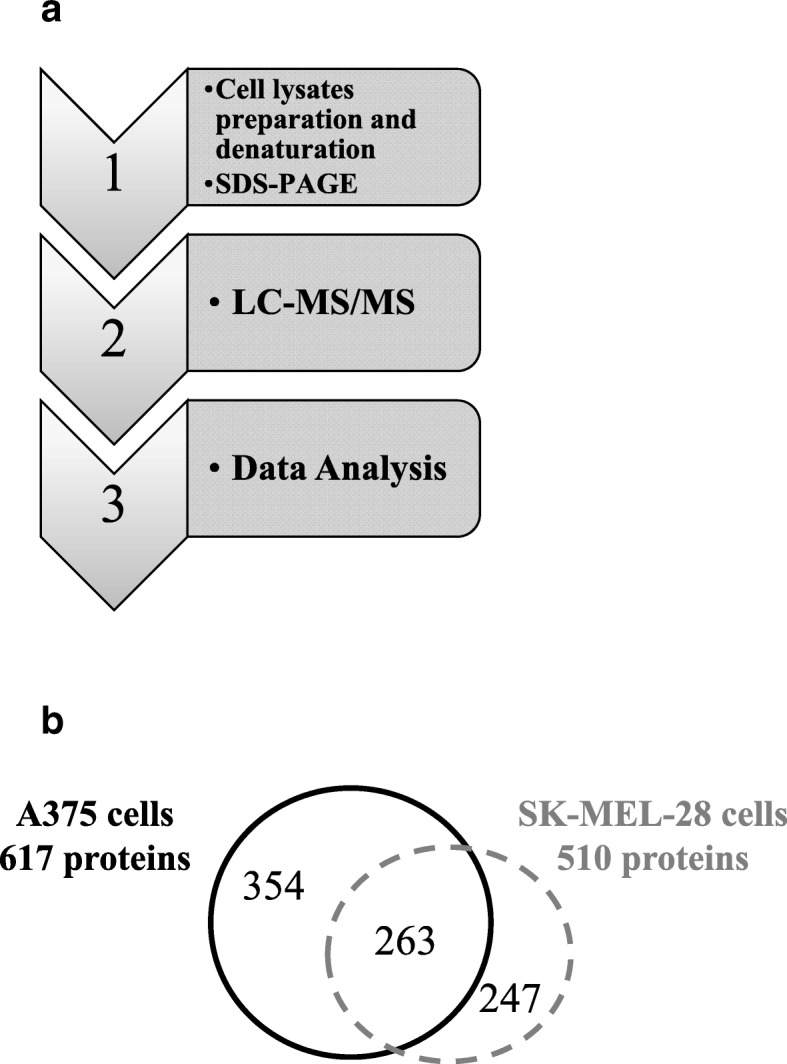
Proteomic Analysis **a** Workflow for the proteomic analysis: proteins analyzed were extracted from cells cultured under serum deprivation conditions. **b** Veen diagram summarizing protein specifically identified in A375 and SK-MEL-28. **c** Functional classification of proteins extracted from cultured cells and identified by proteomic analysis

To further characterize the molecular profile in the two melanoma cells lines, deep proteomics analyses were carried out in A375 and SK-MEL-28 cells extracts according to published protocols [44] and followed by LC-MS/MS. Total number of proteins was calculated according to the workflow reported in Fig. 5a. In SK-MEL-28, 247 “specific” proteins (i.e. not identified in the other cell line extract) out of 510 total proteins were identified, whereas in A375 cells 354 specific proteins out of 617 total proteins were identified; 263 common proteins were identified in the two cell lines (Fig. 5b) and the significantly enriched molecular functions and pathways identified by David software are reported in Additional file 1: Table S4.

The identified specific proteins were analyzed by Gene Ontology and clustering screening through Ingenuity Pathway Analysis (IPA). Functional annotation analysis highlighted in A375 the specific presence of several canonical pathways including VEGF family ligand-receptor interactions, TNFR1 signaling and IL-1 signaling. Such IPA analysis of differentially expressed proteins between A375 and SK-MEL-28 cells identified several “Top Diseases and Functions networks” (Additional file 1: Table S5a and b)potentially involved in melanoma cell aggressiveness (e.g. Cancer, Cellular Assembly and Organization, Cellular Function and Maintenance, Dermatological Diseases and Conditions, Cell Death and Survival, Cellular Development, Cellular Growth and Proliferation, Dermatological Diseases and Conditions, Cellular Assembly and Organization, Cell Cycle, Cellular Movement). The cellular functions highlighted by this IPA analysis on proteomic datasets confirmed that secretory signals might play a role in melanoma aggressiveness.

### Involvement of inflammatory pathways; in vitro validation

**Fig. 6:**
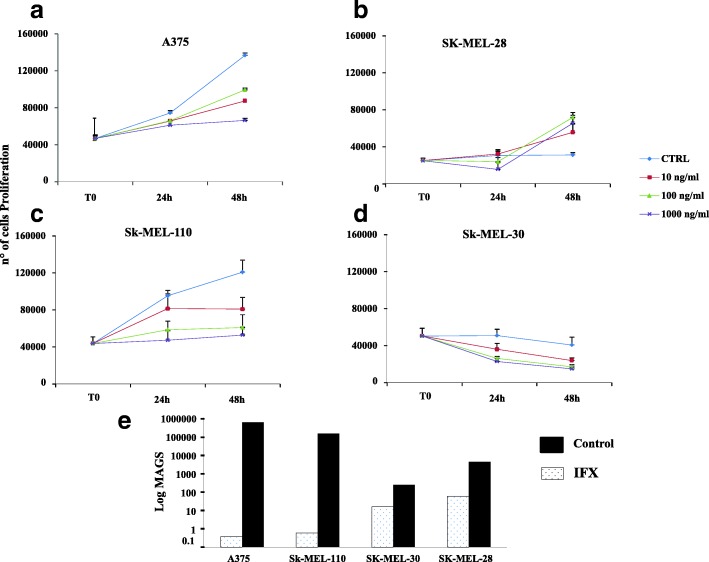
An anti-TNF drug (IFX) affects melanoma cell proliferation rate (**p < 0.001; § *p < 0.0001*). Panel a-d show the anti-proliferative effects of IFX on four different cell lines, while panel E compare the IFX-effects on the MAG scores calculated for the same cells

To achieve a functional validation of a crucial role of TNF-α, cell lines were grown in the presence of increasing doses of Infliximab (IFX), a specific neutralizing TNF antibody. Such treatment decreased significantly A375, SK-MEL-28, SK-MEL-30 and SK-MEL-110 cell proliferation, in a dose- and time-dependent way (Fig. 6a, b, c, d and e). The potential effect of IFX-based anti-TNF treatment was also tested on migratory and invasiveness assays. Results depicted in Fig. 6e show the effect of IFX treatment on the aggressiveness of 4 different human melanoma cells, according to the MAGS scoring system. In all tested cases, IFX treatment reduces by at least 10 times the computed score, and, intriguingly, the melanoma cell lines more sensible to IFX are those showing the highest MAGS under untreated conditions.

### In-patients validation of inflammatory pathways involvement in melanoma compared to nevi

The cytokines or other molecular players found significantly up- or down-expressed in more aggressive melanoma cells compared to less aggressive ones were investigated in transcriptomic datasets available online and derived from biopsies of 45 melanoma patients versus 18 benign nevi (i.e., the GDS1375 dataset in GEO database). The highly significant differences and the consensus with the data obtained on A375 and Sk-MEL-28 cells lines are summarized in Table 4.

**Table 4:** Validation of involved pathways by in silico / in patient analyses

Pathway name and status in A375 vs SK-Mel-28	GEO Analysis (Affymetrix)		
	biopsy of melanomas (n = 45)	biopsy of nevi (n = 18)	t test (melanomas vs nevi)
TIMP3 (Down-regulated, Illumina)	81.64 ± 9.1	92.56 ± 7.23	*p* value 0.00006
IL1RA (Down-regulated, Luminex)	60.02 ± 13.34	73.89 ± 12.15	*p* value 0.00029
VEGFA (Up-regulated, Luminex)	79.31 ± 12.39	60.78 ± 18.15	*p* value 0.00132

Cytokines found significantly modulated in this study by transcriptomics, proteomics and/or secretome analyses comparing A375 vs SK-MEL-28 cells have been matched with transcriptomics analyses from patients biopsies (45 melanomas vs 18 nevi). In this Table the consensus is reported

## Discussion

One of the most critical issues regarding cutaneous melanoma is related to its aggressiveness, which was also related either to mutational and immunological state or anatomical site [4, 50], or to the intrinsic behavior of melanoma cells. To assess the latter point, the proliferation, migration and invasion abilities of ten human melanoma cell lines were studied. To summarize aggressiveness rate of such cancer cells in one unique parameter, the MAG Score was calculated as a single number able to recapitulate proliferation, migration and invasion ability of each melanoma cell. According to these values, it was possible to classify these human melanoma cell lines as high and low aggressive cells. A375 and SK-MEL-28 cells, two of the most studied human melanoma cells, were chosen as model of different aggressiveness and malignancy, also confirmed by analyzing in depth their melanosphere forming capabilities.

**Fig. 7:**
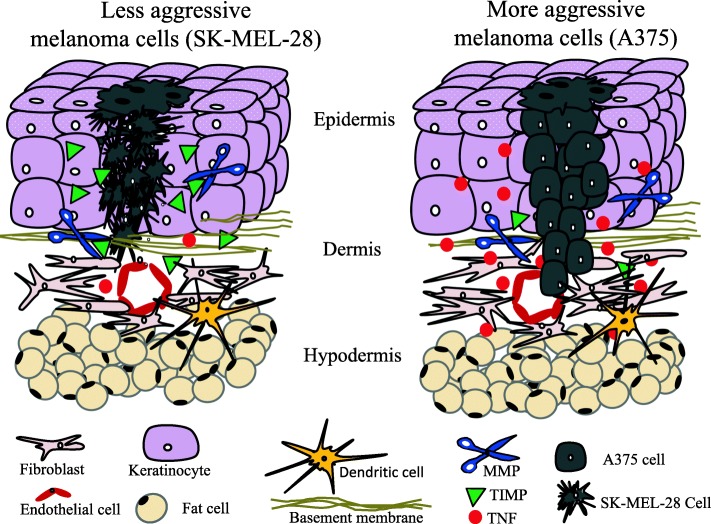
Cartoon depicting molecular mechanisms potentially underlying the TNF/MMPs activity in determining melanoma cell aggressive phenotype

It should be noted that the in vitro aggressive phenotype quantified according to MAGS perfectly matches the in vivo aggressiveness of the two cell lines [51]. This suggests that MAGS may have important clinical applications when patient derived organoids cultures are available. In these cases, the quantitative approach of MAGS may evaluate the organoids aggressiveness for prognostic purposes and to monitor the efficacy of new drugs under development, or drugs combinations, also within a precision medicine framework. In order to identify novel intrinsic molecular pathways responsible for melanoma aggressiveness, the two selected cell lines were analyzed at transcriptomic, proteomic and secretome analysis level. Ingenuity Pathway Analysis from these integrated multiomic investigations highlighted the prominent role of inflammatory response, as well as metalloproteases and secretion of inflammatory cytokines as potentially involved in determining human melanoma cells aggressive phenotype. In the current study, melanoma cells were investigated in vitro without any contact with immune-competent cells, suggesting that intrinsic pathways are likely to be involved in determining their aggressive phenotype. IPA analyses of transcriptomic expression profiles indicated TNF, the MMP-2 and IL-6 pathways as the most significantly upstream regulators, strongly suggesting them as possible key modulators of the melanoma cell aggressiveness. Several pathways resulted particularly dragged into cell aggressive phenotype such as Aryl Hydrocarbon Receptor Signaling; OX40 Signaling Pathway, Antigen Presentation Pathway, Estrogen-mediated S-phase Entry, Cell Cycle, G1/S Checkpoint Regulation, Vitamin D and seleno-proteins, known as potentially important in tumor development and progression. To confirm the results indicating the important role of TNF, MMP and IL-6, the proteomic profile by LC-MS/MS analysis of both cell lines extracts was achieved by applying a multi-denaturation protocol recently developed to increase analytical sensitivity of complex mixtures of proteins [45]. Functional annotation analyses of the collected data revealed a strong implication in aggressiveness traits of post-transcriptional modifications, molecular transport and protein traffic networks and cytokines signaling pathways. Proliferation rates, calculated under serum deprivation conditions and in cells seeded at three different densities, strongly suggested that melanoma cell aggressiveness is related to cell density, highlighting the possibility that a cell-cell interaction crosstalk and/or the secretion of autocrine signals may play a role in melanoma aggressiveness and progression. In the present study, cytokines secretion, evaluated by Luminex technology, showed different levels of pro-inflammatory cytokines like IL-1β, IL-8 and TNF both in melanoma cell lysates and in supernatants. TNF-α showed a higher (more than 20-fold increase) secretion by the most aggressive cell lines. It is noteworthy that TNF has been found involved in the enhancement of tumor invasion partially by upregulating matrix metalloproteases in human skin [52], therefore the low transcript levels of TIMP coupled to the increased enzymatic activity of the MMP2 in more aggressive cells, reported in this study, may be a direct consequence of TNF action, as predicted by IPA. It is important to note that both transcriptional (Illumina - Affymetrix) and bioinformatic (IPA) analyses supported the increased activity of metalloproteases in A375 cells and that such data were perfectly confirmed by the MMP2 enzymatic activity measured. These results were further reinforced by a complementary approach based on analyses of transcriptomic data from biopsies of melanoma patients vs benign nevi, with a consensus within the transcriptomic, proteomic, cytokinomic and zymography data reported in Figs. 3, 4 and 5, in Tables 2, 3 and 4 and in Additional file 1. The hypothesis that TNF-α may be an intrinsic crucial player in melanoma growth and aggressiveness was further tested by inhibiting the TNF secretion through a chimeric monoclonal antibody (INFLIXIMAB-IFX). Upon IFX treatment, the proliferation rate was significantly reduced in 3 out of 4 human melanoma cell lines; the highly aggressive A375 cell line exhibited the lower sensitivity to this drug. The MAG score, based on proliferation, migration and invasion abilities, showed a marked reduction that was very striking for the very aggressive melanoma cell line A375. Recent studies based on mRNA and protein expression show that several MMPs, namely MMP-9, MMP-12 MMP-2, MMP-14, and MMP-19, play a role in melanoma aggressiveness and consequently may represent useful prognostic biomarkers [53–55]. The role of TNF-dependent pathways in melanoma cells growth and malignant phenotype proposed in the current study confirms previous data carried out in similar cellular models [56] as well as the controversial role of TNF in cutaneous melanoma [21]. However, our study suggests, for the first time, a cooperation between MMP-2 enzymatic activity, measured by means of zymography approach, and TNF secretion to define melanoma cells aggressive phenotype, as summarized in Fig. 7. The controversial role of TNF, reported to both inhibit and promote cancer growth, has been explained by the ability of tumor cells to attract TNF-secreting cells through MHC class II molecules expression [21]. Our study investigates expression, secretion and function of molecular signals produced by melanoma cells. A multiomic approach combined with different cellular functions such as proliferation, migration and invasion, lead to develop a new quantitative score called MAGS. In fact, in the present study, for the first time melanoma aggressiveness was assayed by simultaneous multiomic and multifunctional points of view, including enzymatic activities quantification.

Unexpectedly, MMPs mRNA levels were found to be similar in both melanoma cell lines, while bioinformatics analyses indicated that MMPs-related pathways are significantly involved in the phenotypic features of A375 cells (very aggressive) and SK-MEL-28 cells (less aggressive cell type). Such apparently contradictory finding was explained by the functional analysis, which confirmed that, despite similar expression profiles, MMPs enzymatic activity was strongly and significantly different, likely due to the observed different TIMPs expression. Therefore, our findings indicate that the MMPs pathway considered from a functional- rather than just the expression-point of view, may explain, at least in part, the higher A375 cells aggressiveness.

In our in vitro studies, TIMP3 mainly accounts for the observed reduction of TIMPs expression in A375 compared to SK-MEL-28 cells (Fig. 4), strongly matching the in patients validation reported in Table 4, and according to previous studies [57].

Further, we found expression of several cyto- and chemokines to be strongly different in the two cell types, e.g. IP-10 more than 32 times down-modulated, RANTES more than 22 times up-regulated and Eotaxin more than 500 time up-regulated in A375 compared to SK-MEL-28 cells (see Table 2). This signature and the corresponding specific molecular-balance may represent the *scenario* underlying, at least in part, the melanoma aggressiveness. As an example, a significant increase of eotaxin was reported in *humor aqueous* samples from uveal melanoma patients [58], compared to non-melanoma samples, but its involvement has never been reported in aggressive cutaneous melanoma models. Thus, the combined analysis of transcriptomic, proteomic, secretomic and functional data may represent a powerful and novel way to further investigate cancer aggressiveness molecular signatures, as shown in Fig. 7, reporting a simplified model where other important players for melanoma microenvironment and immune response are not taken into account (e.g. lymphocytes and dendritic cells and other molecular signals highly significantly modulated in our study). A crucial concept emerging from the present study is the need to approach complex issues by different and simultaneous functional points of view. The controversial role of TNF [21], as well as TIMPs versatility [58], may be better understood in simplified cellular models carefully characterized in terms of aggressiveness by applying a functional-quantitative approach such as the MAGS reported in this study. It is noteworthy that one of the side effects of long-term therapies with anti-TNF drugs is an increase of cancer risk, including melanoma, or demyelination [27]. The role played by growth factors and cytokines in regulating melanoma cells behaviors was investigated in the past indicating the presence of a complex network with autocrine and paracrine effects [59]. Interestingly, when further investigated at immuno-histochemical level on fresh specimens from melanocytic nevi and primary cutaneous and metastatic melanomas, the expression of some inflammatory mediators and their receptors was found increased with tumor progression [19]. The present study confirmed these findings by more quantitative approaches, indicating that melanoma cell itself secretes large amount of TNF-α, IL-6 and other cytokines, triggering a cascade of effects like, for instance, the increase of MMP2 enzymatic activity, possibly related to the aggressive phenotype of the cell.

## Conclusion

The reported findings indicate i) a novel functional scoring method potentially useful for prognostic purposes and to better characterize cancer cells from patients-derived organoids, ii) new mechanisms underlying melanoma cells aggressiveness and novel molecular targets.

## Additional file

Additional file 1: **Figure S1.** Proliferative rate of A375 and SK-MEL-28 cell lines when cultured at three different cell densities**. Figure S2.** Serum-deprivation induced apoptotic cell death of A375 compared to SK-MEL-28 melanoma cells. **Figure S3**. Effects of MMP2 on downstream transcripts differentially expressed in A357 vs SK-MEL-28 human melanoma cells. **Figure S4**. Effects of TNF on transcripts differentially expressed in A357 vs SK-MEL-28 human melanoma cells. **Figure S5.** Effects of IL6 on downstream transcripts differentially expressed in A357 vs SK-MEL-28 human melanoma cells. **Table S1.** Summary of the culture media in which the different cell lines are grown. **Table S2.** List of transcripts differentially expressed in A375 vs SK-MEL-28 melanoma cell lines. **Table S3**. Ingenuity Pathway Analysis of transcripts differentially expressed in A375 vs SK-MEL-28 melanoma cell lines. **Table S4.** DAVID Analysis of proteins identified in A375 and SK-MEL-28 melanoma cell lines. **Table S5.** Ingenuity Pathway Analysis of proteins identified in A375 and SK-MEL-28 melanoma cell lines. (ZIP 5475 kb)

## Abbreviations

CCL: Chemokine ligands
CM: Cutaneous melanoma
DAVID: Database for annotation, visualization and integrated discovery
DMEM: Dulbecco’s modified Eagle’s medium
EDTA: Ethylene diamine tetra-acetate
ERK: Extracellular signal–regulated kinase
FACS: Fluorescence-activated cell sorting
FBS: Fetal bovine serum
FCS: Fetal calf serum
FGF: Fibroblast growth factor
G-CSF: Granulocyte colony stimulating factor
GM-CSF: Granulocyte-macrophage colony stimulating factor
HPLC: High pressure liquid chromatography
IFN: Interferon
IFX: Infliximab
IL-: Interleukin-
IP-10: Interferon gamma-induced protein 10
IPA: Ingenuity pathway analysis
MAGS: Melanoma aggressiveness score
MCP: Monocyte chemoattractant protein
MEK: Mitogen-activated ERK kinase
MIP: Macrophage inflammatory protein
MMPs: Matrix metalloproteases
nRP-LC-MS/MS: Nano-reversed-phase liquid chromatography tandem mass spectrometry
PBS: Phosphate buffered saline
PDGF: Platelet-derived growth factor
PI: Propidium iodide
PVPF: Polyvinylidene difluoride
RANTES: Regulated on activation normal T cell expressed and secreted.
RNAse A: Ribonuclease A
SD: Standard deviation
TGF-β: Transforming growth factor-beta
TRIDENT: Three denaturation treatment
TIMP: Tissue inhibitor of metalloproteinase
TNF-α: Tumor necrosis factor-alpha
TNFR: Tumor necrosis factor receptor
UR: Upstream regulator
VEGF: Vascular endothelial growth factor
