# Supplementary material for: TNF-alpha and metalloproteases as key players in melanoma cells aggressiveness
Source: J Exp Clin Cancer Res. 2018 Dec 28;37:326. doi: 10.1186/s13046-018-0982-1 (PMC6309098; doi:10.1186/s13046-018-0982-1)
Supplement: Supplementary file 1 — Figure S1. Proliferative rate of A375 and SK-MEL-28 cell lines when cultured at three different cell densities. Figure S2. Serum-deprivation induced apoptotic cell death of A375 compared to SK-MEL-28 melanoma cells. Figure S3. Effects of MMP2 on downstream transcripts differentially expressed in A357 vs SK-MEL-28 human melanoma cells. Figure S4. Effects of TNF on transcripts differentially expressed in A357 vs SK-MEL-28 human melanoma cells. Figure S5. Effects of IL6 on downstream transcripts differentially expressed in A357 vs SK-MEL-28 human melanoma cells. Table S1. Summary of the culture media in which the different cell lines are grown. Table S2. List of transcripts differentially expressed in A375 vs SK-MEL-28 melanoma cell lines. Table S3. Ingenuity Pathway Analysis of transcripts differentially expressed in A375 vs SK-MEL-28 melanoma cell lines. Table S4. DAVID Analysis of proteins identified in A375 and SK-MEL-28 melanoma cell lines. Table S5. Ingenuity Pathway Analysis of proteins identified in A375 and SK-MEL-28 melanoma cell lines. (ZIP 5475 kb) [file 13046_2018_982_MOESM1_ESM.zip › Additional file 1 Table S1 Rossi et al.docx]

| Cell type | Growth media composition |
| --- | --- |
| Human Melanocyte | MBM - 4 Basal medium  +  rh-Insulin  L-glutamine  Epineprhine  Calcium Cloride  Peptide growth factor  M8 supplement |
| Human Melanoma Cell Line |  |
|  |  |
| A375 | DMEM Basal Medium + 10% FBS + L-glutamine |
|  |  |
| A375M | DMEM Basal Medium + 10% FBS + L-glutamine |
|  |  |
| ME665 | DMEM Basal Medium + 10% FBS + L-glutamine |
|  |  |
| Mel 397 | DMEM Basal Medium + 10% FBS + L-glutamine |
|  |  |
| MEL 501 | DMEM Basal Medium + 10% FBS + L-glutamine |
|  |  |
| MeWo | DMEM Basal Medium + 10% FBS + L-glutamine |
|  |  |
| Preyer | DMEM Basal Medium + 10% FBS + L-glutamine |
|  |  |
| SK-Me-110 | RPMI Basal Medium + 10% FBS + L-glutamine |
|  |  |
| SK-MEL-28 | RPMI Basal Medium + 10% FBS + L-glutamine |
|  |  |
| SK-Mel-30 | RPMI Basal Medium + 10% FBS + L-glutamine |
