# Supplementary material for: TNF-alpha and metalloproteases as key players in melanoma cells aggressiveness
Source: J Exp Clin Cancer Res. 2018 Dec 28;37:326. doi: 10.1186/s13046-018-0982-1 (PMC6309098; doi:10.1186/s13046-018-0982-1)
Supplement: Supplementary file 1 — Figure S1. Proliferative rate of A375 and SK-MEL-28 cell lines when cultured at three different cell densities. Figure S2. Serum-deprivation induced apoptotic cell death of A375 compared to SK-MEL-28 melanoma cells. Figure S3. Effects of MMP2 on downstream transcripts differentially expressed in A357 vs SK-MEL-28 human melanoma cells. Figure S4. Effects of TNF on transcripts differentially expressed in A357 vs SK-MEL-28 human melanoma cells. Figure S5. Effects of IL6 on downstream transcripts differentially expressed in A357 vs SK-MEL-28 human melanoma cells. Table S1. Summary of the culture media in which the different cell lines are grown. Table S2. List of transcripts differentially expressed in A375 vs SK-MEL-28 melanoma cell lines. Table S3. Ingenuity Pathway Analysis of transcripts differentially expressed in A375 vs SK-MEL-28 melanoma cell lines. Table S4. DAVID Analysis of proteins identified in A375 and SK-MEL-28 melanoma cell lines. Table S5. Ingenuity Pathway Analysis of proteins identified in A375 and SK-MEL-28 melanoma cell lines. (ZIP 5475 kb) [file 13046_2018_982_MOESM1_ESM.zip › Additional File Figures S1-S5 Rossi_et_al_.docx]

**ADDITIONAL FILE**

*Rossi et Al.*

**Figures S1-S5**

**Figure legends**

**Figure S1**

**
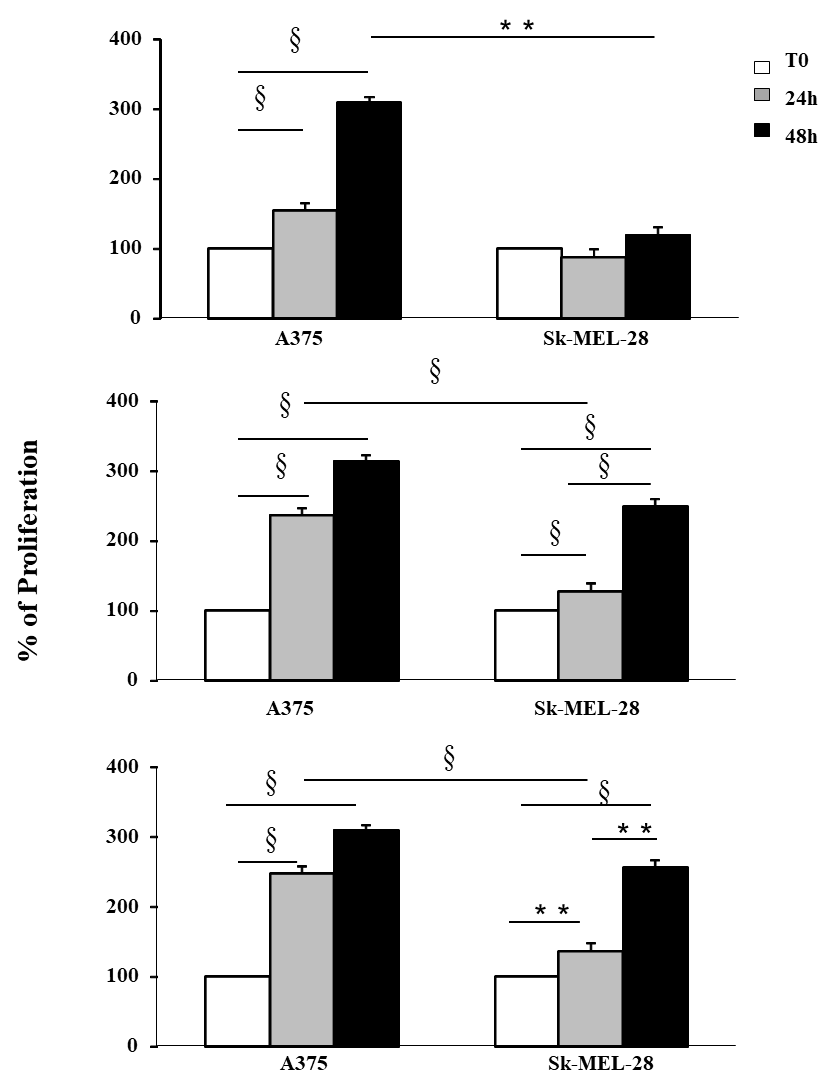
**

**Figure S2**

**
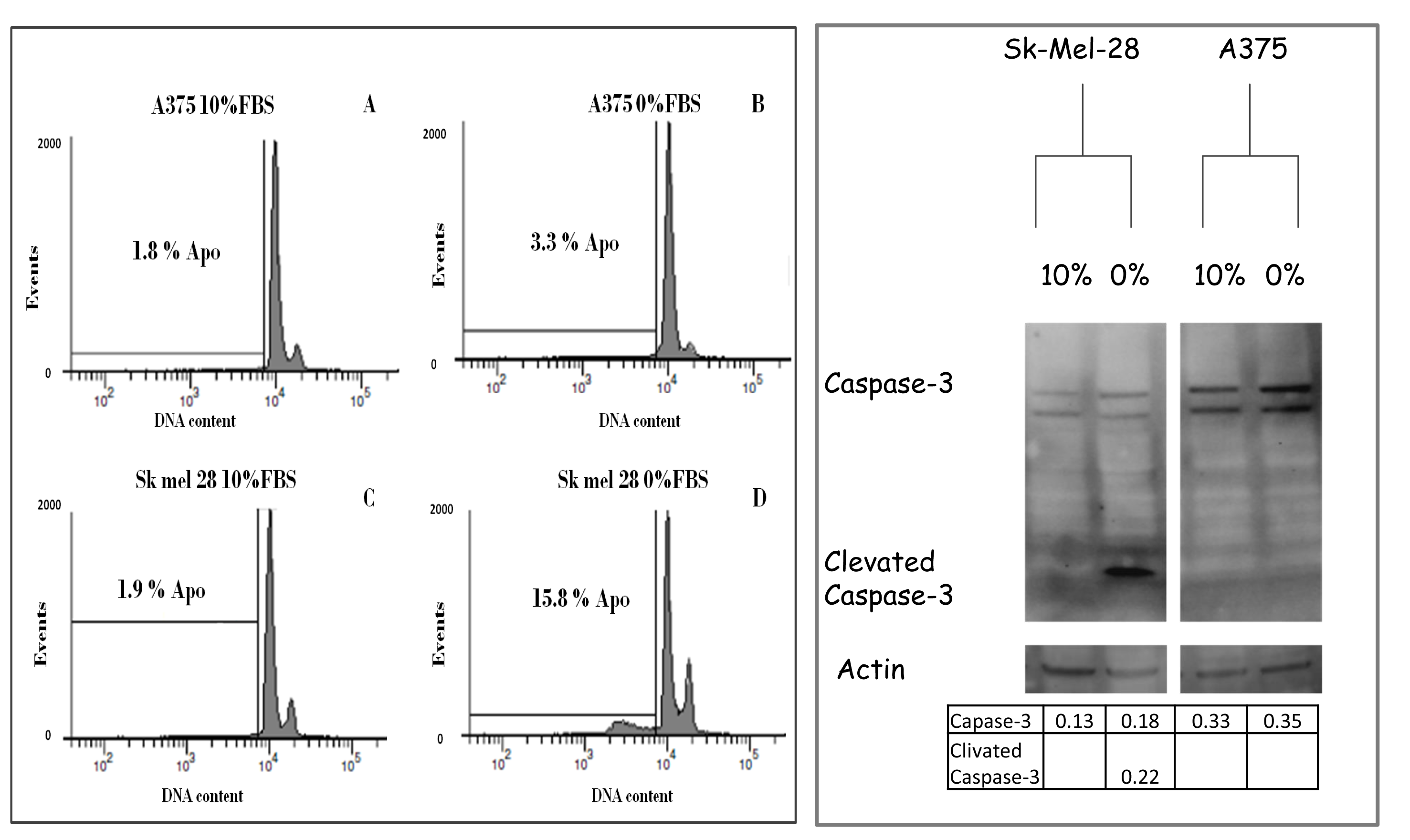
 Figure S3**

**
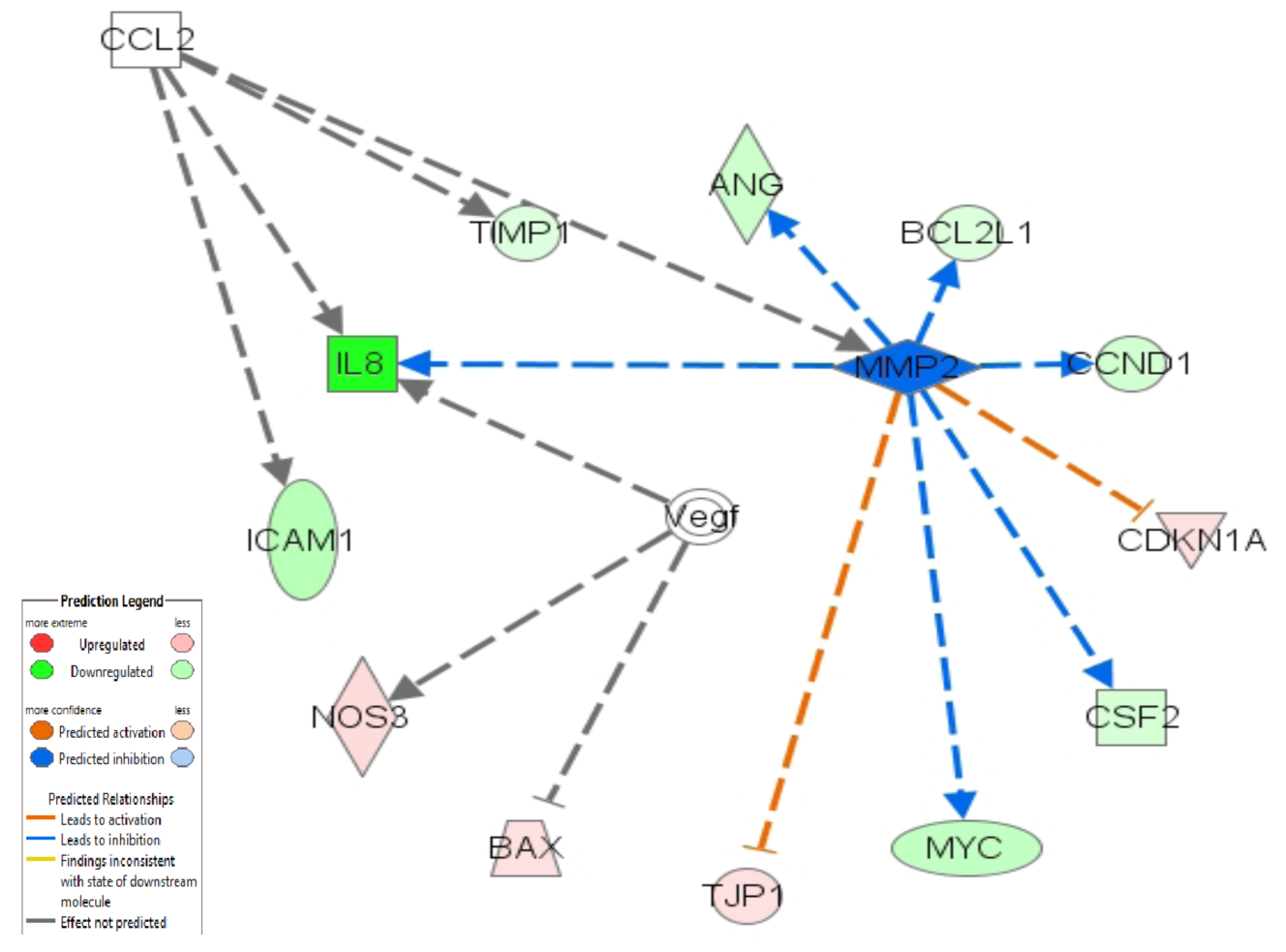
 Figure S4**

**
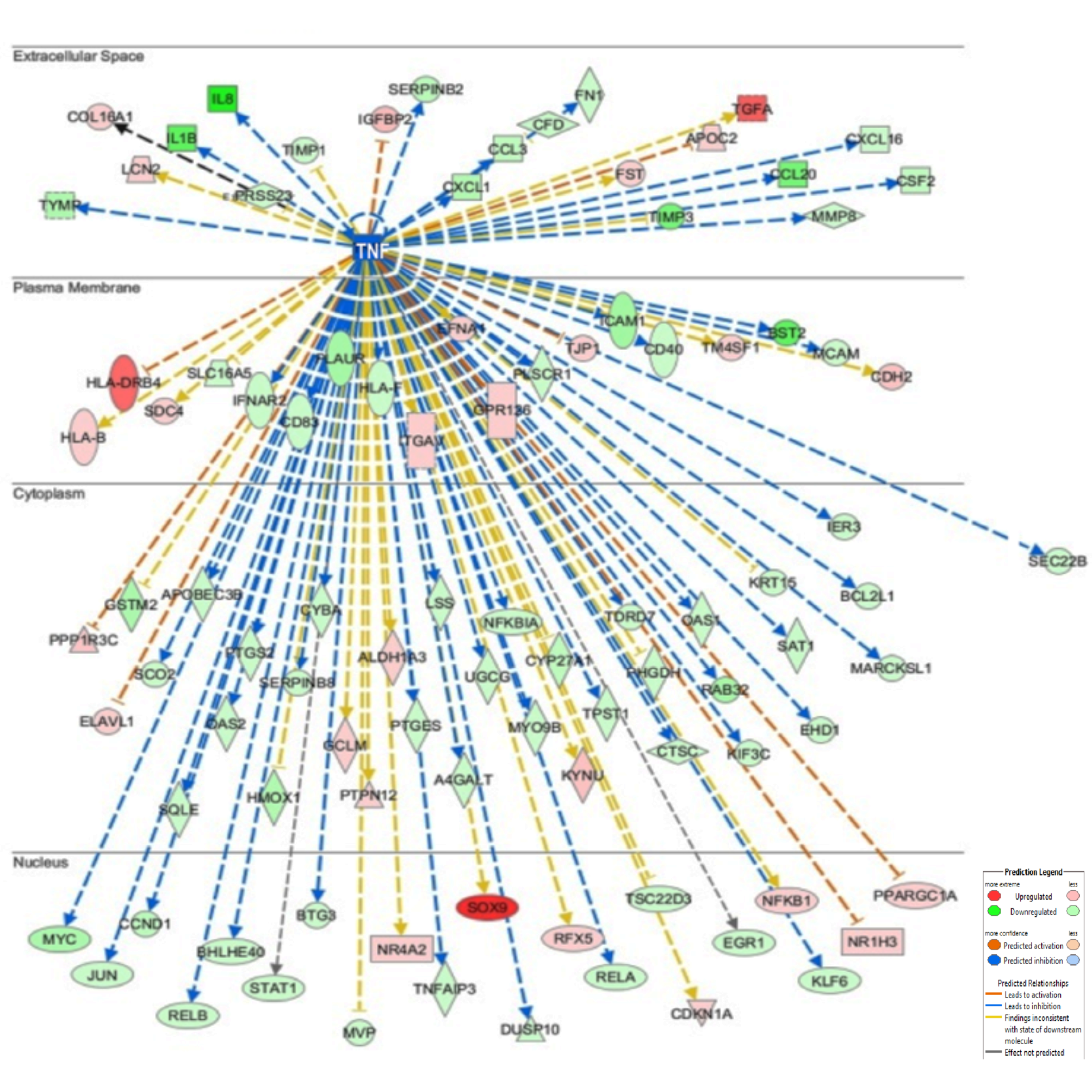
**

**Figure S5**

**
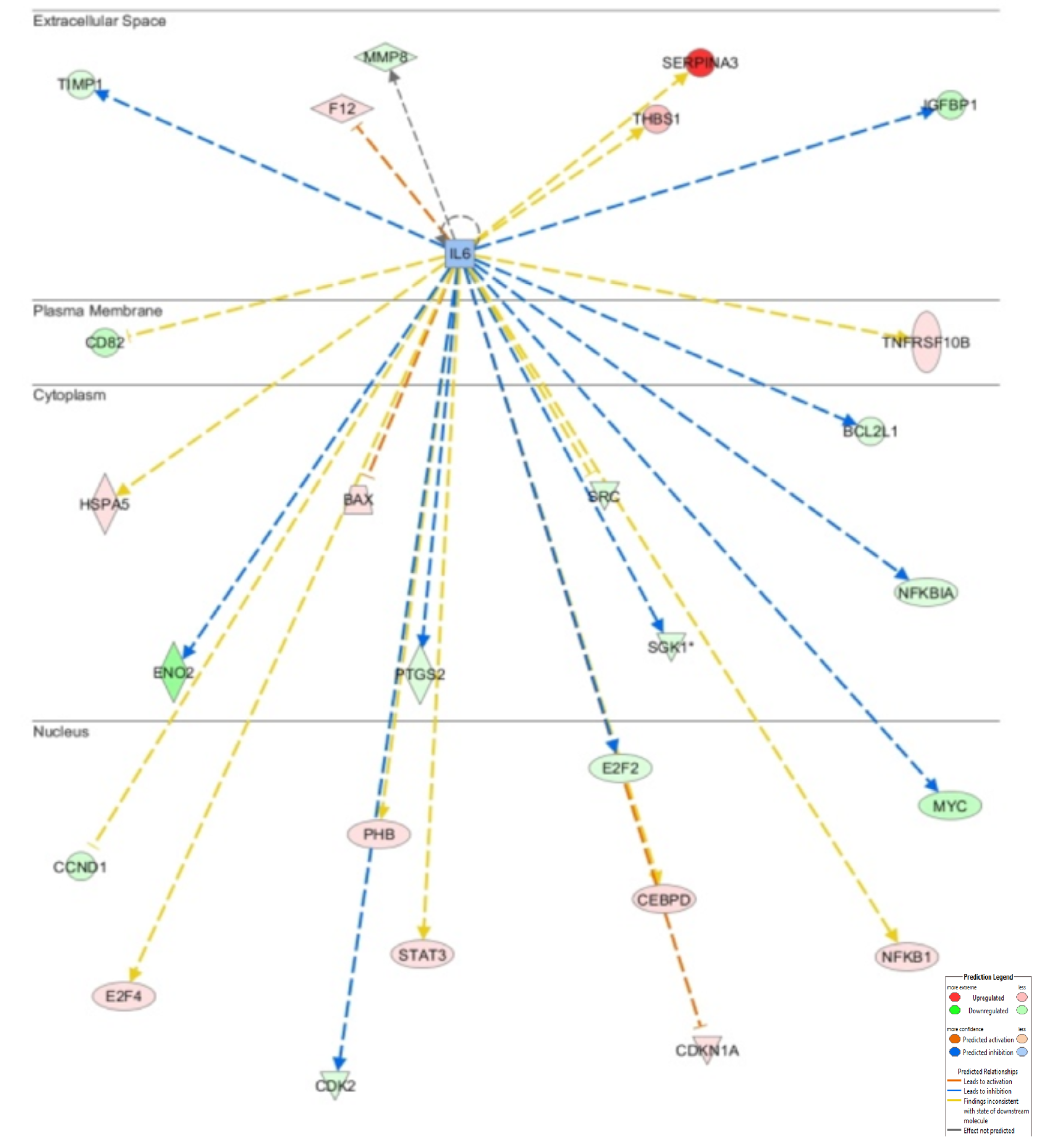
**

**Additional Figure legends**

**Figure S1**. Proliferative rate of A375 and SK-MEL-28 cell lines when cultured at three different cell densities, namely very low (upper panel), intermediate and high density (lower panel). Cells were grown in serum-free medium and cell number was measured after 24 and 48 h. White box = time zero, grey box = 24 hours, black box = 48 hours. Cells were plated as reported under Methods (Section Proliferation assay). Data shown are means of three independent experiments that are expressed as percentage of proliferation ( ** P < 0.001; §P < 0.0001).

**Figure S2.** Serum-deprivation induced apoptotic cell death of A375 compared to SK-MEL-28 melanoma cells.

**Figure S3.** Effects of MMP2 on downstream transcripts differentially expressed in A357 *vs* SK-MEL-28 human melanoma cells.

**Figure S4.** Effets of TNF on transcripts differentially expressed in A357 *vs* SK-MEL-28 human melanoma cells.

**Figure S5.** Effects of IL6 on downstream transcripts differentially expressed in A357 *vs* SK-MEL-28 human melanoma cells.
